# Depletion of regulatory T cells in ongoing paracoccidioidomycosis rescues protective Th1/Th17 immunity and prevents fatal disease outcome

**DOI:** 10.1038/s41598-018-35037-8

**Published:** 2018-11-08

**Authors:** Nayane A. L. Galdino, Flávio V. Loures, Eliseu F. de Araújo, Tania A. da Costa, Nycolas W. Preite, Vera Lúcia G. Calich

**Affiliations:** 1 Departamento de Imunologia, Instituto de Ciências Biomédicas, Universidade de São Paulo, São Paulo, SP Brazil; 2 Present Address: Instituto de Ciência e Tecnologia, Universidade Federal de São Paulo, São José dos Campos, SP Brazil

## Abstract

In human paracoccidioidomycosis (PCM), a primary fungal infection typically diagnosed when the disease is already established, regulatory T cells (Treg) cells are associated with disease severity. Experimental studies in pulmonary PCM confirmed the detrimental role of these cells, but in most studies, Tregs were depleted prior to or early during infection. These facts led us to study the effects of Treg cell depletion using a model of ongoing PCM. Therefore, Treg cell depletion was achieved by treatment of transgenic C57BL/6^DTR/eGFP^ (DEREG) mice with diphtheria toxin (DT) after 3 weeks of intratracheal infection with 1 × 10^6^ *Paracoccidioides brasiliensis* yeasts. At weeks 6 and 10 post-infection, DT-treated DEREG mice showed a reduced number of Treg cells associated with decreased fungal burdens in the lungs, liver and spleen, reduced tissue pathology and mortality. Additionally, an increased influx of activated CD4^+^ and CD8^+^ T cells into the lungs and elevated production of Th1/Th17 cytokines was observed in DT-treated mice. Altogether, our data demonstrate for the first time that Treg cell depletion in ongoing PCM rescues infected hosts from progressive and potentially fatal PCM; furthermore, our data indicate that controlling Treg cells could be explored as a novel immunotherapeutic procedure.

## Introduction

Regulatory T cells (Treg cells) are a fundamental component in regulation of innate and adaptive immune responses. These cells play an essential role in self-tolerance maintenance, anti-tumor response, transplantation immunity and infectious processes control^1–3^. In their regulatory function, Treg cells can exert protective or deleterious effects depending on the experimental setting or disease process. By suppressing excessive immunity, Tregs can function protectively by restraining tissue damage caused by uncontrolled inflammation; however, the suppression of immunity can lead to uncontrolled pathogen growth and disease progression that is deleterious to the host.

There are several T cell subsets that possess regulatory activity. Naturally occurring Treg cells are CD4^+^ T cells that mature in the thymus and constitutively express CD25 (the alpha chain of IL-2R), low levels of CD45RB, and Foxp3 a transcription factor that is fundamental in the preservation of peripheral tolerance^4^. Induced Treg cells can be generated from conventional T cells under certain defined microenvironments such as the presence of TGF-β and retinoic acid^5,6^. In addition to CD25 (IL-2Rα), Treg cells express other activation markers such as CTLA-4 (CD152, cytotoxic T lymphocyte-associated antigen 4), GITR (glucocorticoid-induced tumor necrosis factor-receptor-related protein), OX40 (CD134), and L-selectin (also known as CD62 ligand, CD62L)^7,8^. In addition to the aforementioned markers, Treg cells also possess enhanced expression of Neuropilin-1, CD39, CD73, Helios and CCR5^9,10^.

The suppressive activity of Treg cells can be mediated by inhibitory cytokines, metabolic interference, cytolysis, and modulation of dendritic cell function. A set of inhibitory cytokines -TGF-β, IL-10, and IL-35- are released under Treg cell stimulation and may inhibit the function of both innate and effector T cells. This inhibition can affect pro-inflammatory mechanisms mediated by Th1, Th2 and Th17 responses^11–13^.

The presence and the modulatory function of Treg cells have been described in experimental models and human fungal infections, including paracoccidioidomycosis, which is the most prevalent systemic mycosis in Latin America. An infection with *P. brasiliensis* can present three outcomes: 1) an asymptomatic infection identified by positive delayed-type hypersensitivity (DTH) skin tests, but no symptoms of the disease; 2) the acute/subacute form is characterized by rapid fungal dissemination and involvement of the lymph nodes, liver, spleen and bone marrow; and, 3) the chronic form presenting heterogeneous clinical manifestations, ranging from unifocal to multifocal forms^14–16^. The acute form of PCM is distinguished by predominant Th2/Th9 cell activation. Patients with the chronic form develop a mixed immune response with the predominant differentiation of Th17/Th22 cells, high production of IL-17 and IL-22, and variable amounts of Th1 and Th2 cytokines^16^. In contrast, individuals with asymptomatic infection develop a prevalent Th1 immunity^16,17^.

The characteristic immunosuppression observed in PCM patients has been associated with elevated numbers of Foxp3 expressing Treg cells within lesions and blood^16,18–20^. Furthermore, circulating CD4^+^CD25^+^FoxP3^+^ cells of PCM patients can exhibit high surface expression of molecules associated with Treg function such as CTLA-4, LAP-1 (latency-associated peptide (TGF-β)), and GITR. Treg cells isolated from peripheral blood of PCM patients revealed that both contact-dependent suppression and production of soluble factors can be part of their function^18,19^.

An initial study by our group demonstrated that Treg cells exert a deleterious effect on mice resistant (A/J) and susceptible (B10.A) to *P. brasiliensis* infection. Depletion of Treg cells by an anti-CD25 monoclonal antibody led to less severe and regressive infection, in addition to decreased tissue pathology in both mouse strains^21^. Further studies in the murine model provided evidence for the dual role of Treg cells in the severity of pulmonary PCM^22^. Using a loss- and gain-of-function experimental approach for the manipulation of Treg cells *in vivo*, Treg cells were shown to be both protective and detrimental to pulmonary PCM. The transfer of Tregs combined with CD4^+^Foxp3^−^ T cells to SCID mice generated the more efficient and well-adjusted immune response able to limit pathogen growth and excessive tissue inflammation, leading to reduced disease severity and high survival rates^22^. Nevertheless, in human PCM all published studies have associated the presence of Treg cells with the severe forms of the disease. However, it is also known that the improvement of some PCM patients is only achieved when the anti-fungal therapy is associated with anti-inflammatory drugs, highlighting the importance of controlled inflammation as a therapeutic tool^14,23^. Altogether, these findings indicate that further studies on the role of Tregs in PCM are necessary to better understand the immunopathology of this disease.

Most studies on the role of Treg cells in human and experimental PCM have revealed the detrimental effect Treg cells on protective adaptive immunity. Importantly, all experimental studies were performed using the depletion of Treg cells at the onset of infection, but the immunomodulatory effect of Treg cells depletion in the ongoing disease has yet to be addressed. Because the human PCM is diagnosed late when the disease is already well established, we thought it would be important to characterize the effect of Treg cells depletion in ongoing PCM. C57BL/6 mice transgenic for the expression of diphtheria toxin receptor (DTR) and GFP (“green fluorescent protein”) under the influence of the FoxP3 transcription factor gene, which is characteristic of Treg cells, were here employed. The depletion of Treg cells in ongoing PCM significantly reduced disease severity by increasing effective Th1/Th17 immunity able to reduce fungal loads and consequent pathogen mediated tissue pathology. More importantly, these findings open a perspective of immunotherapeutic treatment for established PCM by controlling the number and/or function of Treg cells.

## Results

### Determination of the ideal dose of DT to deplete Treg cells and kinetics of Tregs recovery

Initially, we determined the dose of DT that efficiently depletes Treg cells from DEREG mice using a previously described protocol^24^ with minor modifications. The doses of 1.0, 0.5 or 0.25 µg of DT or PBS were injected i.p. for two consecutive days and DEREG mice sacrificed 12 h later. Lung leukocytes were obtained and labelled with anti-CD25 and anti-Foxp3 antibodies. As shown in Fig. S1A,B, the frequencies of GFP^+^ cells and CD25^+^Foxp3^+^ Treg cells were determined by flow cytometry using two different gate strategies. The cytometric analysis of lung leukocytes from DEREG mice showed that 1.0 and 0.5 µg of DT, but not with PBS, caused a profound depletion of Treg cells as demonstrated by the reduced presence of pulmonary GFP^+^ and CD4^+^CD25^+^Foxp3^+^ cells (Fig. S1C). Accordingly, the treatment of control C57BL/5 WT mice with 1.0 µg of DT did not reduce the frequency of these cells (Fig. S1D). This assay led us to choose the dose of 0.5 µg of DT to deplete Treg cells of DEREG mice.

**Figure 1:**
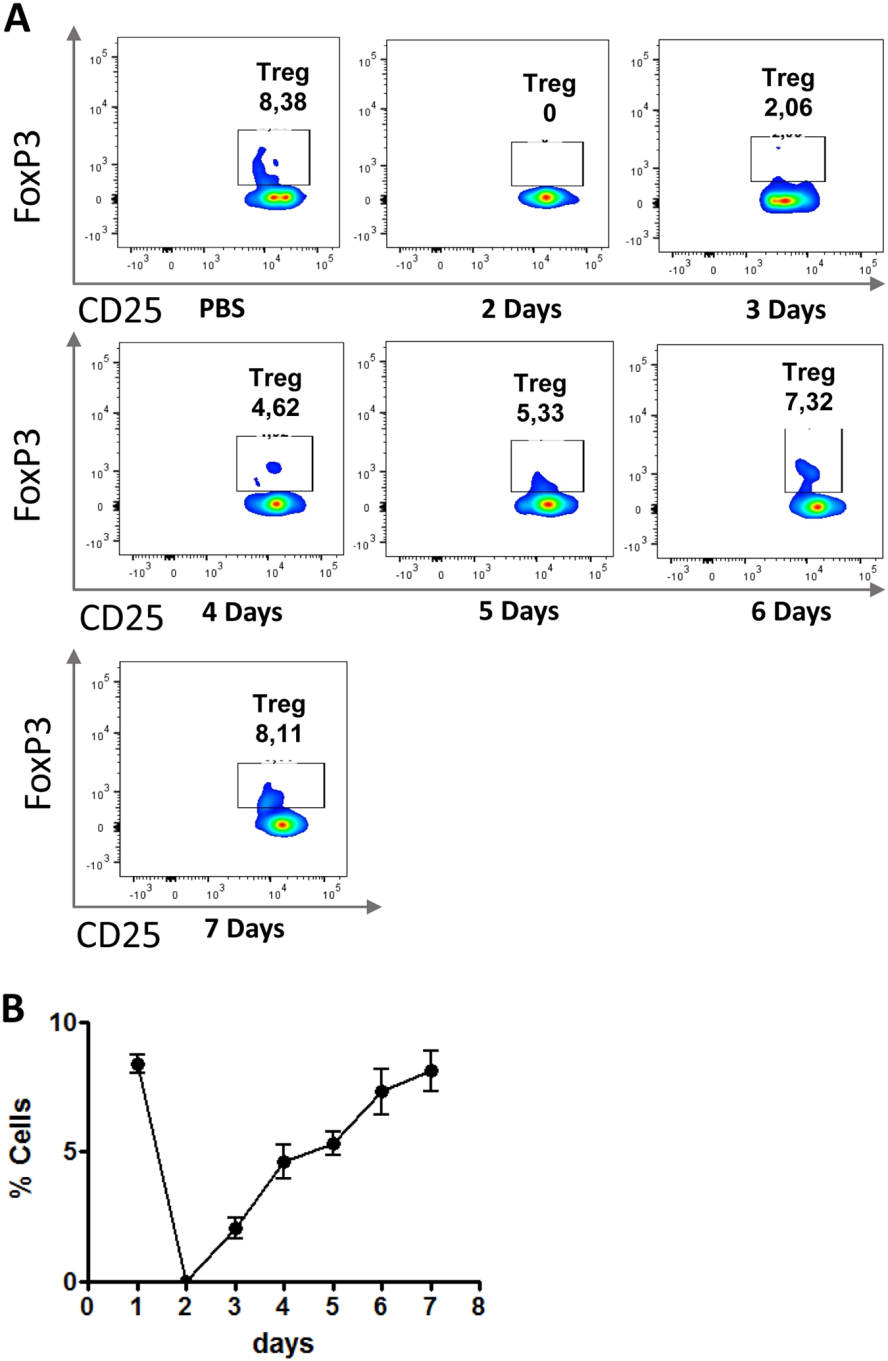
Recovery of Treg cells after treatment with 0.5 µg of DT for two consecutive days. DEREG mice (n = 4–5) were treated for two consecutive days with 0.5 µg of DT and the frequency of Treg cells in the blood was followed in the next 7 days. The frequency of CD4^+^Foxp3^+^ cells was measured by flow cytometry. As can be seen, Treg cells returned to normal frequency at the day 7 of treatment (**A**,**B**). Treg cells reached the lowest frequency at day 2 after treatment and progressive recovery their level in the next 5 days (**B**). The experiment was repeated 3 times with similar results.

We have then evaluated the recovery of Treg cells during a 7 days period after treatment with 0.5 µg of DT for two consecutive days. The gate strategy used to determine Treg cells is shown in the Fig. S1. It can be seen that at day 2 no GFP^+^CD25^+^Foxp3^+^ cells were found in the lungs, but the frequency of these cells increased in the following days and reached normal levels at day 7 after treatment (Fig. 1A,B). This result led us to use a weekly treatment to sustain Treg depletion in the course of the infection.

### Depletion of Treg cells of DEREG mice does not cause inflammatory damage of organs

It is well known that absence of Treg cells is associated with severe autoimmune pathologies^1^. This fact led us to investigate if the here used protocol to deplete Treg cells for 10 weeks would cause inflammatory damage to several organs. Thus, mice were treated weekly with two consecutive doses of 0.5 µg of DT, and at week 10 their organs were obtained and histologically analyzed. As depicted in Fig. S2 no changes or inflammatory infiltrates were seen in the spleen, brain, heart, liver lung and kidney of DT-treated DEREG mice, indicating the absence of autoimmune reactivity.

### Depletion of Treg cells in the ongoing disease reduces the fungal loads, tissue pathology and mortality rates of DEREG mice

**Figure 2:**
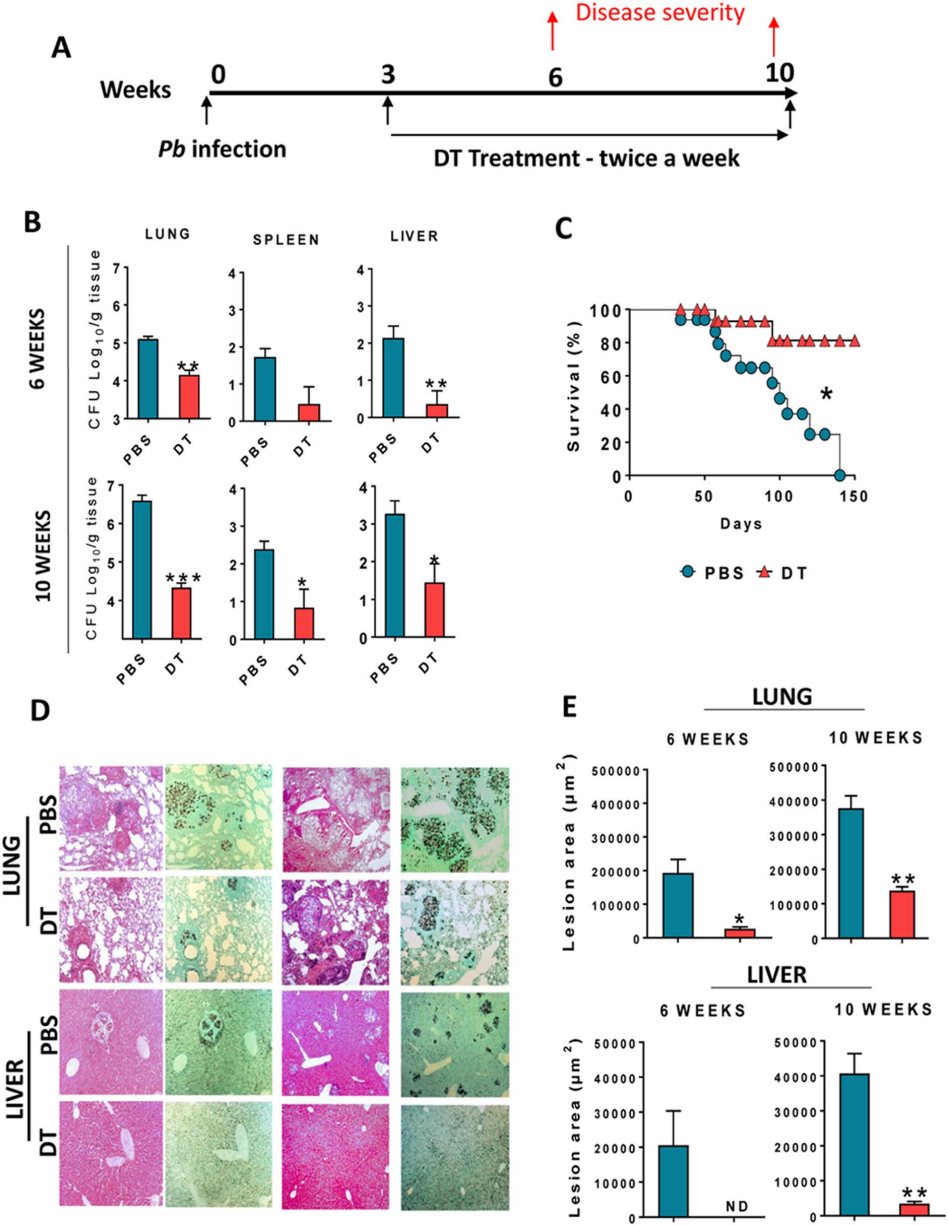
Depletion of Treg cell in ongoing PCM leads to decreased fungal burdens, tissue pathology and mortality rates of DEREG mice. Groups (n = 6) of DEREG mice were i.t. inoculated with 1 × 10^6^ *P. brasiliensis* yeast cells. Three weeks later they were treated with two consecutive daily doses/week of 0.5 µg of DT or PBS by the i.p. route for next 3 or 7 weeks (**A**). At weeks 6 and 10 after infection mice were sacrificed and their lungs, liver and spleen assessed for the presence of viable fungal cells using a CFU assay (**B**). Survival curves of DT or PBS treated infected DEREG mice were determined in a period of 160 days (**C**). Mice were sacrificed at weeks 6 and 10 after infection and their organs analyzed by histology for the presence of inflammation (HE, left panels) and fungal cells (Grocot, right panels) (**D**). The total area of lung and liver lesions was calculated at weeks 6 and 10 after infection (**E**). Experiments were repeated twice, and bars represent mean ± SEM (*p < 0.05, **p < 0.005 and ***p < 0.001).

DEREG mice were infected i.t. with 1 × 10^6^ *P. brasiliensis* yeasts. Three weeks after infection, infected mice were treated twice weekly with 0.5 µg of DT or PBS and the treatment was maintained until the 6^th^ and 10^th^ weeks after infection (Fig. 2A). At these time points, mice were sacrificed, and their organs assessed for the presence of viable fungal cells. As seen in Fig. 2B, compared with control PBS treated mice, DT treatment led to reduced fungal burdens in the lungs, spleen and liver of DEREG infected mice. The histologic sections of lungs and livers from DT or PBS treated DEREG mice were analyzed for the presence of inflammation (HE) and fungal cells (Grocott, methamine silver staining). It can be seen (Fig. 2D) that DT-treated DEREG mice showed diminished inflammatory reactions accompanied by reduced fungal loads in both organs and post-infection periods analyzed. The lesion areas of lung and liver of DT or PBS treated mice were measured and a significant reduction was observed in Treg depleted mice (Fig. 2E). The reduced fungal burdens and tissue damage led to reduced mortality of DT-treated mice (Fig. 2C). These data led us to conclude that the reduction of Treg cells in ongoing PCM exerts a protective effect to infected hosts.

### DT treatment of infected DEREG mice causes increased influx of CD4^+^CD25^+^Foxp3^−^ T cells concomitantly with decreased presence of CD4^+^CD25^+^Foxp3^+^ Treg cells

**Figure 3:**
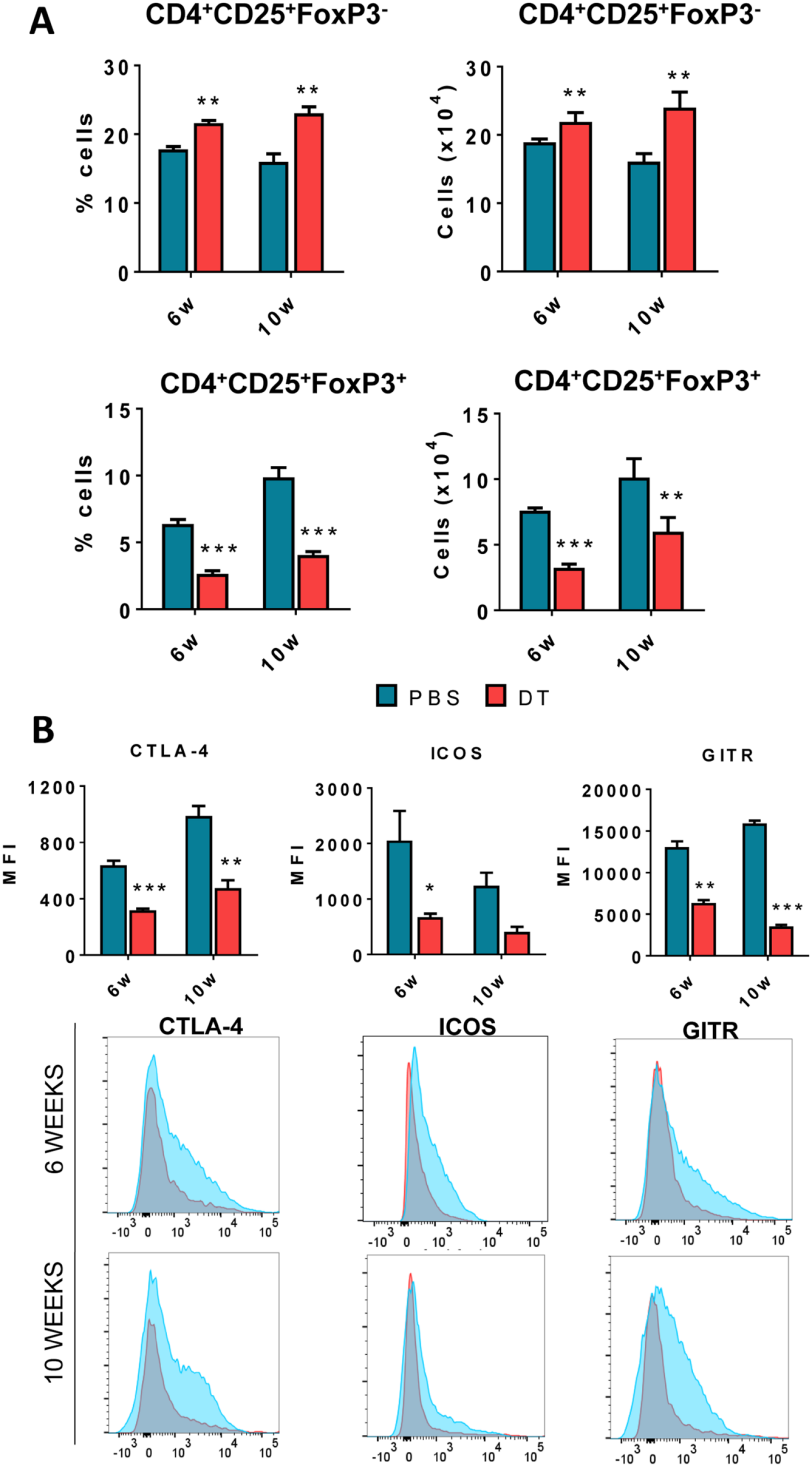
Diphtheria toxin treatment of DEREG mice in ongoing PCM reduces the number of CD4^+^Foxp3^+^ Treg cells and the expression of Treg cells markers. The phenotypic analysis of CD4^+^Foxp3^−^ and CD4^+^Foxp3^+^ T cells of lung infiltrating leukocytes from DT-treated and control (PBS treated) DEREG mice was performed by flow cytometry at weeks 6 and 10 week after *P.brasiliensis* infection. The lung cells were obtained as described in Material and Methods and labeled with antibodies conjugated to different fluorochromes. The lung infiltrating leukocytes were gated by FSC/SSC analysis. The cells were gated for CD4 and then for Foxp3 expression (**A**). Treg cells markers (CTLA-4, ICOS and GITR) were then assessed in CD4^+^Foxp3^+^ T cells (**B**). One hundred thousand cells were acquired on FACS CANTO II and subsequently analyzed by FlowJo software. Data are expressed as means ± SEM of three independent experiments using 5 mice per group (**p < 0.005 and ***p < 0.001).

The lungs of DT-treated and control DEREG infected mice were obtained at weeks 6 and 10 after infection. The lung infiltrating leukocytes were isolated, and the expression of T cell markers evaluated by flow cytometry. The gate strategy used is depicted in Fig. S3. DT-treated mice showed an increase influx of CD4^+^Foxp3^−^ T cells allied with decreased numbers of CD4^+^Foxp3^+^ Treg cells into the lungs (Fig. 3A). When CD4^+^Foxp3^+^ Treg cell markers were evaluated, it was seen a decreased expression of CTLA-4, ICOS and GITR indicating a lower activation of these cells present in the lung cell infiltrates (Fig. 3B).

### Depletion of Treg cells in ongoing PCM induces increased influx of activated macrophages, dendritic cells and CD4^+^ and CD8^+^ T lymphocytes into the lungs of DEREG infected mice

**Figure 4:**
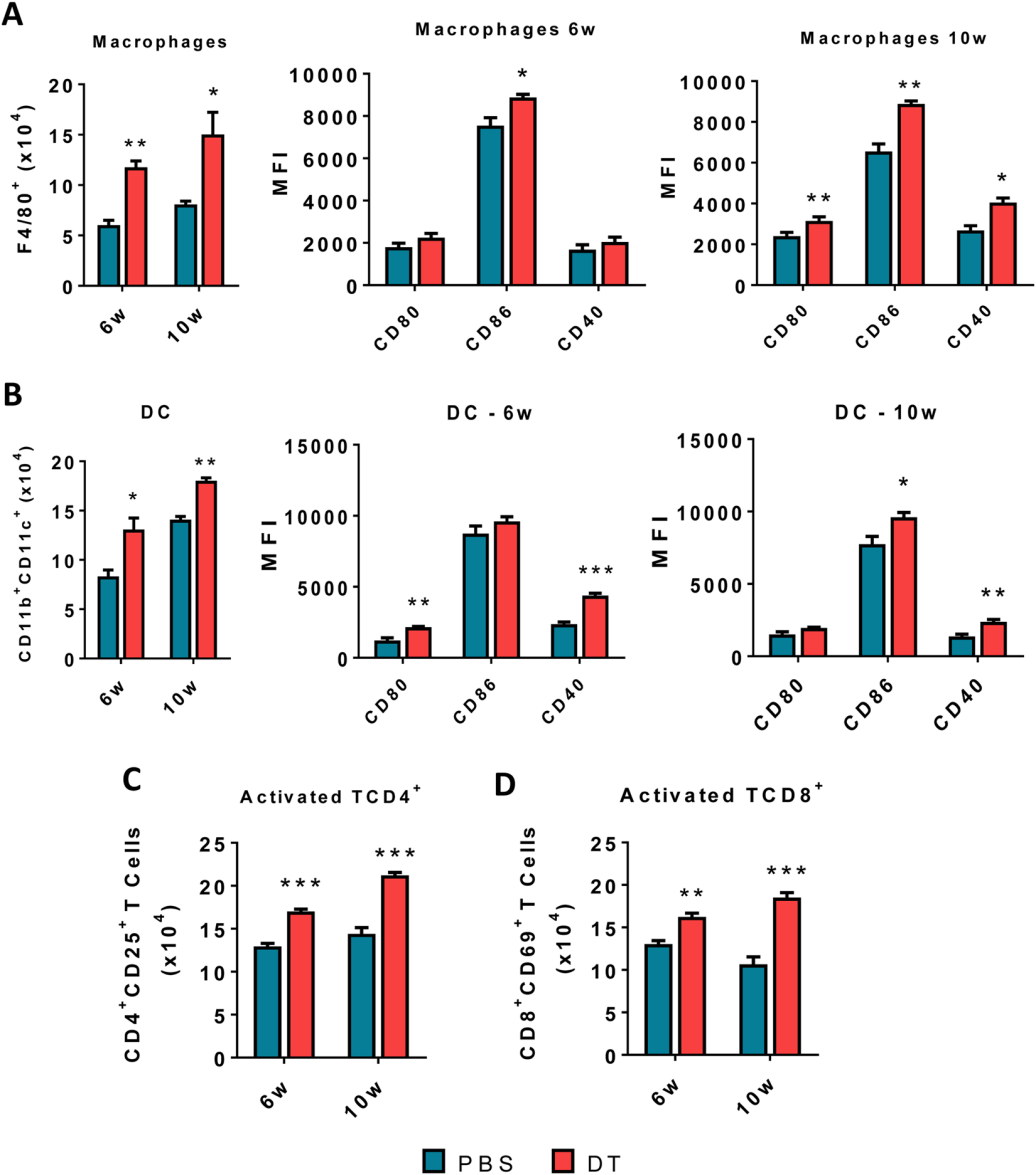
DT treatment of DEREG mice in ongoing PCM increases the number and activation of macrophages, DCs and T cells that migrate to the site of lesion. The phenotypic analysis of macrophages (CD11c^+^F4/80^+^) and dendritic cells (CD11b^+^CD11c^+^) cells and some activation markers (CD80, CD86, CD40) of lung infiltrating leukocytes from DT-treated and control (PBS treated) DEREG mice was performed at weeks 6 and 10 week after *P.brasiliensis* infection (**A**,**B**). The lung cells were obtained as described in Material and Methods and labeled with antibodies conjugated to different fluorochromes. The lung infiltrating leukocytes were gated by FSC/SSC analysis. The cells were gated for CD11c/CD11b expression and then the CD11b cells were characterized for F4/80 expression. Macrophages and DC markers (CD80, CD86 and CD40) were then assessed in both cell subpopulations (**A**,**B**). For T cells phenotyping the lung infiltrating leukocytes were gated by FSC/SSC analysis and then gated for CD4^+^ or CD8^+^ expression. The expression of CD25 and CD69 activation markers were then evaluated in CD4^+^ and CD8^+^ T cells, respectively. One hundred thousand cells were acquired on FACS CANTO II and subsequently analyzed by FlowJo software. Data are expressed as means ± SEM of three independent experiments using 5 mice per group (*p < 0.05, **p < 0.005 and ***p < 0.001).

**Figure 5:**
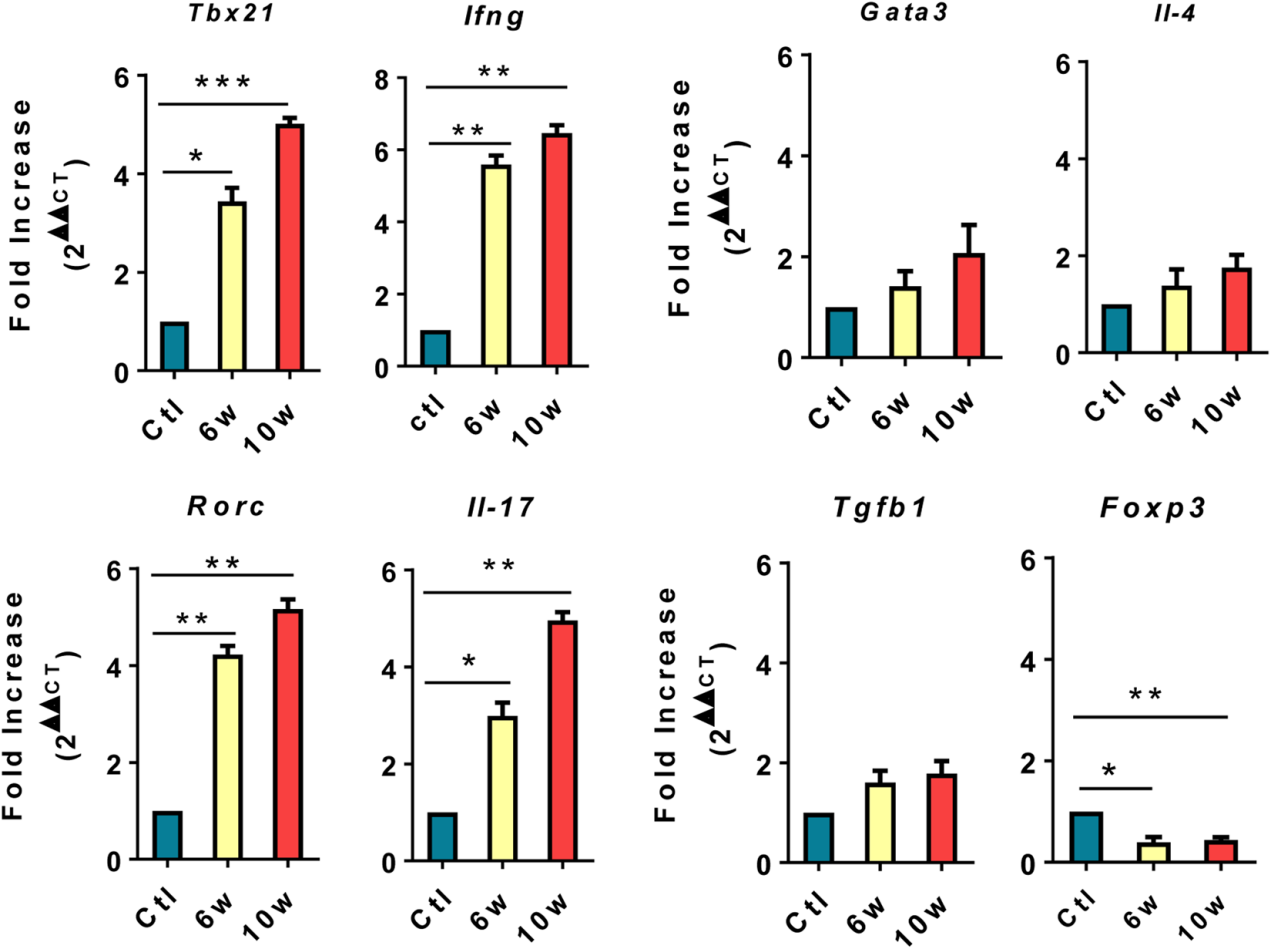
Depletion of Treg cells in ongoing PCM of DEREG mice increases the gene expression of cytokines and transcription factors of Th1 and Th17 cells concomitant with reduced Foxp3 expression. T helper (Th) cells are characterized by different cytokines and master transcription factors profiles which are used to define their subsets. Th1 cells preferentially secrete IFN-γ and express the transcription factor Tbx21. Th2 cells that differentiate under the influence of many cytokines typically secrete IL-4, IL-5, IL-9 and IL-13 under the control of GATA3 transcription factor. The combination of TGF-β and IL-6 as well as IL-1β and IL-23 influence the differentiation of Th17 cells which have RORγτ as the master transcription factor and IL-17 as the major synthesized cytokine. In addition, TGF-β causes the expression of Foxp3 transcription factor that leads to the differentiation of Treg cells that secrete TGF-β, IL-10 and IL-35. Here is shown the relative expression of mRNA of IFN-γ, IL-4, IL-17, TGF-β, Tbx21, Gata3, Rorc and Foxp3 in whole lung cells of DT and PBS (control) treated DEREG mice after 6 and 10 weeks of *P. brasiliensis* infection. The level of gene transcription was determined by Real-Time PCR. Bars show mean ± SEM from three independent experiments using at least four mice per group (*p < 0.05, **p < 0.01 and ***p < 0.001).

The number and activation of F4/80^+^ macrophages, CD11b^+^CD11c^+^ dendritic cells (DCs) as well as CD4^+^CD25^+^ and CD8^+^CD69^+^ T lymphocytes were determined by flow cytometry in DT-treated and control DEREG mice at weeks 6 and 10 of infection. The gate strategies used are shown in the Figs S3 and S4. Macrophages and DCs appeared in increased numbers in Treg depleted in comparison with control mice (Fig. 4A,B). Furthermore, macrophages increased the expression of activation markers (CD80, CD86 and CD40) that was more evident at week 10 after infection (Fig. 4A) while DCs of Treg depleted mice increased the expression of CD86 and CD40 at week 6 and CD80 and CD40 at week 10 after infection (Fig. 4B). Importantly, at both periods of infection an increased number of activated CD4^+^CD25^+^ and CD8^+^CD69^+^ T cells were found in the lungs of Treg depleted mice (Figs 4C and 5D).

### Treg cells depleted mice showed increased mRNA expression for transcription factors and cytokines of Th1 and Th17 lymphocytes allied with reduced expression of Foxp3

T helper (Th) cells are characterized by different cytokines and master transcription factors profiles which are used to define their subsets. Th1 cells preferentially secrete IFN-γ and IL-2, express the transcription factor T-bet and are induced by several mediators including IL-12 and IL-18. Th2 cells that differentiate under the influence of many cytokines including IL-4, IL-33, IL-25 and thymic stromal lymphopoietin, typically secrete IL-4, IL-5, IL-9 and IL-13 under the control of GATA3 transcription factor. The combination of TGF-β and IL-6 as well as IL-1β and IL-23 influence the differentiation of Th17 cells which have RORγτ as the master transcription factor and IL-17 as the major synthesized cytokine. On the other hand, TGF-β causes the expression of Foxp3 transcription factor that leads to the differentiation of Treg cells that secrete TGF-β, IL-10 and IL-35^25^.

To further characterize the effect of Treg cells depletion on T cell subsets, the mRNA expression for transcription factors and cytokines of Th1, Th2, Th17 and Treg cells was measured by RT-PCR in lung leukocytes obtained from DT and PBS (control) treated infected mice at weeks 6 and 10 post infection. As shown in Fig. 5, both, the mRNA for the transcription factor (*Tbx21*) and cytokine (*ifng*) which characterize Th1 cells appeared in increased levels at weeks 6 and 10 after infection. No differences were found in *Gata3* and *IL-4* mRNA expression indicating that Th2 cells were not significantly altered. In contrast, both, the mRNA for the transcription factor (*Rorc*) and the typical cytokine (*IL-17*) of Th17 cells were produced in increased amounts by Treg depleted mice at both periods of the disease. Interestingly, no differences in the mRNA of TGF-β but decreased levels of Foxp3 mRNA were observed in DT-treated mice at both post-infection periods studied (Fig. 5).

### Treg cells depletion in ongoing PCM increased the presence of Th1 and Th17 cells in lung inflammatory infiltrates

**Figure 6:**
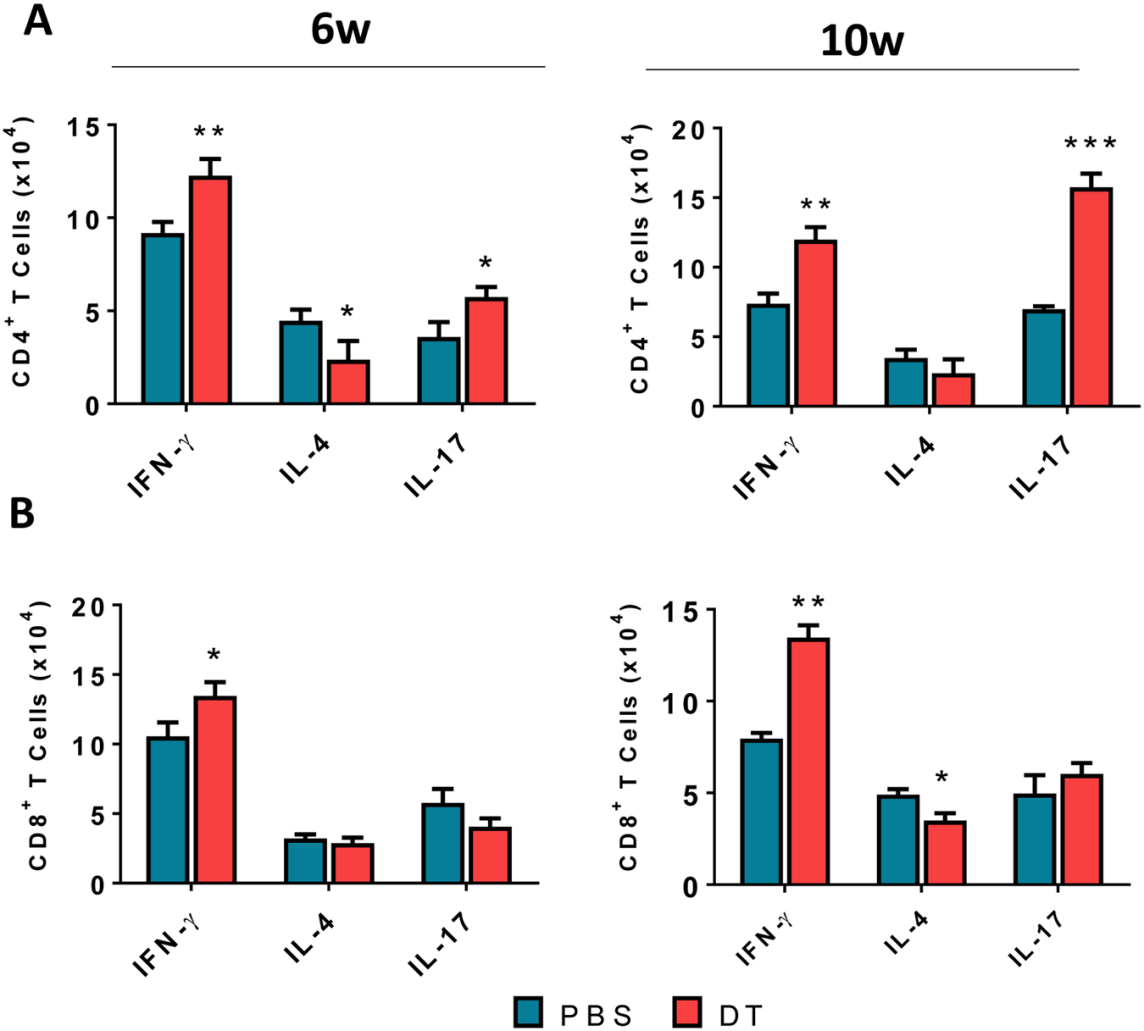
The reduction of Treg cells in ongoing PCM of DEREG mice increases the number of pulmonary Th1 and Th17 cells but reduces the presence of Th2 lymphocytes. The phenotypic analysis of lung infiltrating lymphocytes from DT and PBS (control) treated DEREG mice was performed at weeks 6 and 10 of *P. brasiliensis* infection. The lung cells were obtained as described in Material and Methods and labeled with antibodies conjugated to different fluorochromes. The lung infiltrating leukocytes were gated by FSC/SSC analysis. The cells were gated for CD4^+^ (**A**) or CD8 (**B**) expression and then for intracellular expression of IFN-γ, IL-4 and IL-17. One hundred thousand cells were acquired on FACS CANTO II and subsequently analyzed by FlowJo software. Data are expressed as means ± SEM of three independent experiments using 5 mice per group (*p < 0.05, **p < 0.01 and ***p < 0.001).

Lung infiltrating leukocytes from DT-treated and untreated DEREG mice were obtained at weeks 6 and 10 after infection and intracellular presence of IFN-γ^+^, IL-4^+^ and IL-17^+^ in CD4^+^ and CD8^+^ T cells was assessed by flow cytometry. The gate strategy used to evaluate these T cell subsets is shown in Fig. S3. An increased presence of IFN-γ^+^ and IL-17^+^ CD4^+^ T cells was seen at weeks 6 and 10 after infection, while only at the first period studied IL4^+^ CD4^+^ T cells appeared in decreased numbers (Fig. 6A). This indicated that reduction in Treg cells resulted in prevalent Th1/Th17 responses associated with reduced Th2 immunity. A significant increase in IFN-γ^+^ CD8^+^ lymphocytes was detected at weeks 6 and 10, and a reduction in CD8^+^ T cells expressing IL-4 was seen at week 10 post-infection in Treg depleted mice. No differences in IL-17^+^CD8^+^ T cells were detected (Fig. 6B).

### Treg cells depletion in ongoing PCM increases the Th1/Th17 cytokines and reduces Treg cells-associated cytokines

**Figure 7:**
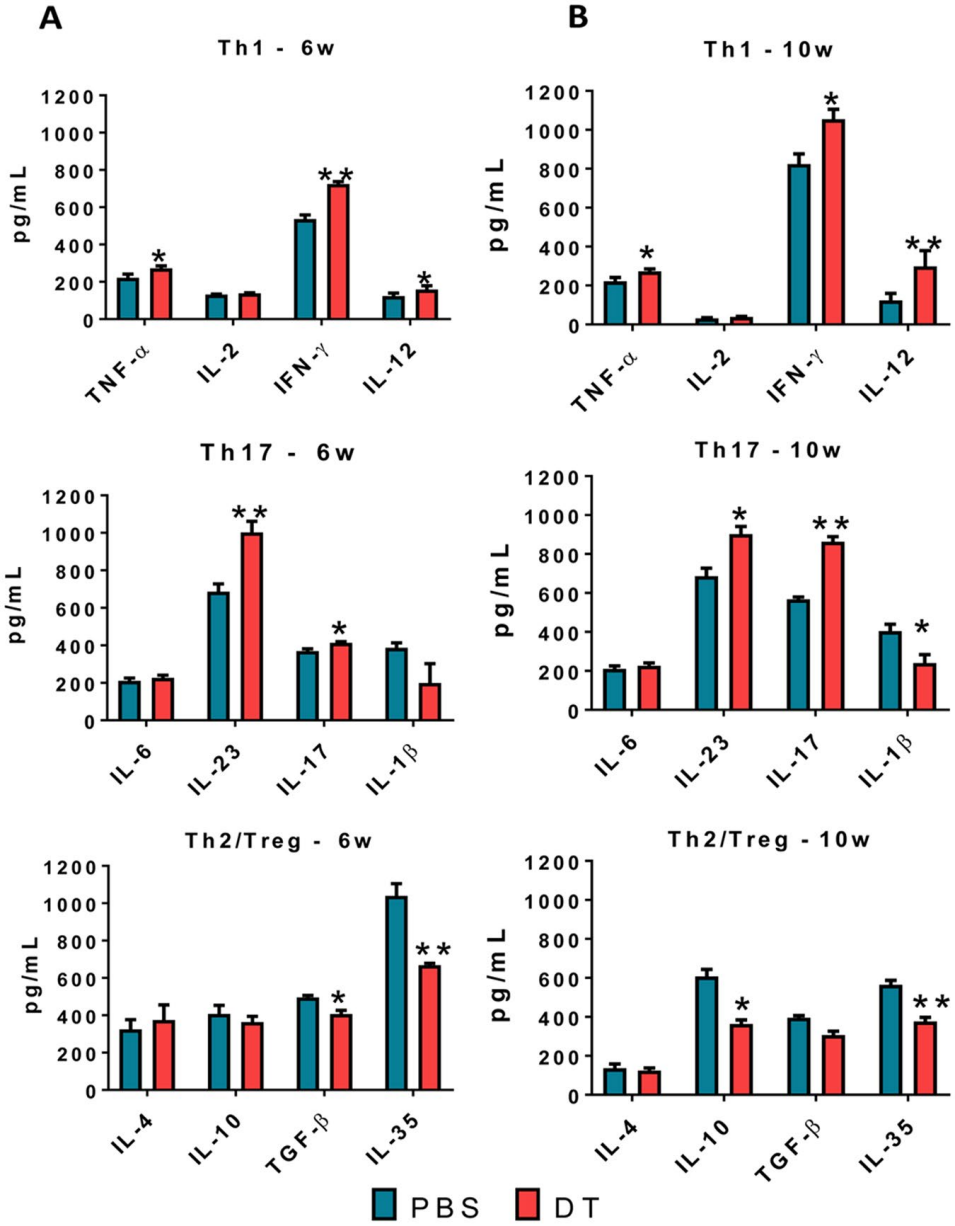
The reduction of Treg cells in ongoing PCM of DEREG mice controls the levels of pulmonary cytokines. Cytokines were measured by ELISA in lung homogenates of DT and PBS (control) treated DEREG mice at weeks 6 and 10 of *P. brasiliensis* infection. Bars show mean ± SEM of two independent experiments using 5 mice per group (*p < 0.05, **p < 0.01).

Lung cell supernatants from DT and PBS treated infected DEREG mice were obtained at weeks 6 and 10 of infection and pro- and anti-inflammatory cytokines measured by ELISA (Fig. 7). Increased levels of TNF-α, IFN-γ, IL-12, IL-23 and IL-17 was concomitant with reduced levels of TGF-β and IL-35 at both periods of infection assayed. Diminished levels of IL-10 were also seen at week 10 post-infection. These data indicate that Treg cells depletion in ongoing PCM caused a prevalent Th1/Th17 response controlled by reduced levels of anti-inflammatory cytokines produced by Treg cells.

Cytokines were also measured by ELISA in the supernatants of macerated spleens and livers of DT-treated and untreated DEREG mice obtained at weeks 6 and 10 weeks after infection (Fig. S5A,B). At week 6 post-infection, increased levels of TNF-α, IFN-γ, IL-12, IL-23 and IL-17 were found in the liver and spleen supernatants associated with reduced levels of TGF-β only in the spleens. At week 10, Th1 and Th17 cytokines (IFN-γ, IL-12, IL-17) were present in higher levels in the spleen and liver supernatants. At this time point, IL-10 and TGF-β were reduced in the spleens whereas IL-35 was the only Treg cytokine that appeared in reduced levels in the liver supernatants (Fig. S5A,B).

## Discussion

Using an experimental approach that allows the adequate manipulation of Treg cells *in vivo*, it was possible to demonstrate that the reduction of these cells in ongoing pulmonary PCM is beneficial to the course of the disease. The existence of DEREG transgenic mice expressing the diphtheria toxin receptor under the control of the Foxp3 gene makes it possible to deplete the Treg subpopulation at any disease stage. The vast majority of experimental studies on the function of Treg cells employed anti-CD25 antibodies inoculated before or at the onset of infection. It is well known that this antibody is useful to selectively deplete Treg cells prior to the immune system activation, due high CD25 expression by these cells. After the infection, the antibody cannot be used due to the increased number of activated T cells expressing high levels of CD25. Therefore, the DEREG model of infection allowed us to demonstrate the beneficial effects of Treg cells depletion during the course of PCM. This is particularly relevant to human PCM that is diagnosed when the disease is well established.

Initially, we verified that 99.98% of the Treg cells could be depleted by using diphtheria toxin (DT) inoculated by the i.p. route at a concentration of 0.5 μg/mL. As previously shown by Lahl *et al*.^24^, our examination of the kinetics of Treg cell reconstitution demonstrated the recovery of pulmonary Treg cells within 7 days following DT treatment in uninfected mice. Importantly, our depletion protocol did not cause autoimmunity-related pathology with severe lymphadenopathy and splenomegaly, as described by Lahal *et al*.^26^ using daily DT treatment of DEREG mice for 20 consecutive days. Therefore, we were able to confirm previous studies^27,28^ showing that a small number of residual Treg cells after DT treatment is sufficient to avoid deleterious autoimmunity. In addition, the depletion protocol used here^24^ was sufficient to reduce Treg cells even at the chronic phase and alter disease outcome without causing autoimmunity or excessive tissue pathology. As expected, DT treatment resulted in Treg cells depletion associated with increased expansion of CD4^+^Foxp3^−^ cells reflecting the suppressive function of Tregs on T cell subpopulations. In addition to reducing the number of Treg cells, DT treatment also led to a decrease expression of membrane molecules (GITR, ICOS and CTLA-4) associated with the suppressor or deactivating function of Treg lymphocytes. Similar effect was observed in other infection models, such as tuberculosis and a viral infection^29,30^.

Foxp3 has been considered a lineage-specific transcription factor of Treg cells specialized in the suppression of immunity^1,4^. However, several studies have indicated that Foxp3 expression might not be restricted to immunosuppressive CD4^+^CD25^+^Foxp3^+^ Treg cells. Some CD8^+^ T, invariant NKT, B, epithelial and tumor cells could potentially express Foxp3, although not necessarily presenting suppressive activity^31^. The expression of Foxp3 by myeloid cells is controversial, and in DEREG mice Mayer *et al*.^32^ have not identified Foxp3^+^ macrophages. In our study we have not characterized the expression of Foxp3 in lymphoid or myeloid cells except in CD4^+^ T lymphocytes implicating that other Foxp3^+^ cells might have contributed to the findings here reported. However, our previous studies^21,22^ clearly indicated the detrimental effects of excessive CD4^+^CD25^+^Foxp3^+^ Treg cells in pulmonary PCM leading us to postulate that the protective effects here observed was due to the reduced number of this T cell subset induced by DT treatment of DEREG mice.

At both, the sixth and tenth weeks post-infection, a reduction in fungal loads was observed in the target organs, demonstrating the inhibitory function of Treg cells on pathogen clearance by host immunity. This result is consistent with our previous findings^21,22^ and other previous reports^33^, demonstrating that Treg cells favor fungal growth in the lesions. The reduced fungal loads paralleled the decreased number and size of lesions indicating that fungal-mediated inflammation and pathology is highly relevant in pulmonary PCM.

Treg cells can exert protective or detrimental roles in different infectious processes. For example, depletion of Treg cells by anti-CD25 antibodies augmented the parasitic burden and mortality of *Toxoplasma gondii* infected mice^34^. In *Cryptococcus neoformans* infection, DT treatment of DEREG mice resulted in increased inflammatory lung injury and exacerbated Th2-type response^35^. In contrast, the findings reported here and those recently reported by our group, using C57BL/6 Foxp3^GFP^, resistant A/J and susceptible B10.A mice, show that Treg cells were deleterious to pulmonary PCM^21,22^.

In addition to the rescue of protective T cell immunity mediated by the increased presence of activated CD4^+^ and CD8^+^ T cells at the site of infection, a higher number of activated macrophages and dendritic cells was also observed; this indicates the contribution of activated CD4^+^ and CD8^+^ T cells to the control of fungal growth and severity of the disease developed by DT-treated mice. This finding is consistent with those observed in other infections such as those caused by *Plasmodium yoelii, Leishmania spp*. and *M. tuberculosis* where the depletion of Treg cells has also resulted in increased activation of T cell immunity^29,36–38^.

As previously shown by Lankford and Frunch^39^, the increase in IL-12 and IL-23 cytokines in the lungs, not only contributed to the activation of T cells, but also possibly contributed to the activation of macrophages, NK and dendritic cells of the DT-treated mice; this activation improves their effector and co-stimulatory function. The quantification of cytokines has also demonstrated that DT-treated DEREG mice produce less pulmonary IL-10 than their controls at weeks 6 and 10 post-infection. Because *P. brasiliensis* infected IL-10^−/−^ mice present a more efficient control of the disease than WT mice^40^, the lower levels of IL-10 observed were potentially important to the increased resistance of DT-treated mice. Simultaneously, the increase in IL-17 and IFN-γ indicated an improved Th1 and Th17 immune response. This cytokine profile is consistent with the increased mRNA expression of Tbet and RORC transcription factors as well as IFN-γ and IL-17 mRNAs seen in DT-treated DEREG mice. DT treatment reduced the levels of IL-1β in lung supernatants. We attribute this unexpected finding to the reduced fungal loads induced by Treg cells depletion. Our previous studies have shown that IL-1β is synthesized following NLRP3 inflammasome activation induced by TLRs and dectin-1 recognition of *P. brasiliensis* components^41^. Therefore, the diminished fungal loads observed at weeks 6 and 10 post-infection have possibly triggered diminished macrophages and dendritic cells NLRP3 activation and reduced IL-1β production. It is well known that IL-1β can contribute to Th17 differentiation but here the increase IL-17 differentiation was not accompanied by increased levels of this cytokine. We can suppose, however, that the improved Th17 differentiation has occurred at early periods of treatment and/or due to a new balance of other microenvironmental cytokines such as IL-6, TGF-β and IL-23 have contributed to Th17 expansion. No significant differences in the expression of IL-4 and GATA-3 mRNA as well as secreted IL-4 were detected. However, the reduced presence of CD4^+^ and CD8^+^ T lymphocytes presenting intracellular IL-4 was observed in the lungs of DT-treated mice. These facts indicate that Treg cells contribute to reduce pro-inflammatory Th1/Th17 lymphocytes and to facilitate Th2 expansion in pulmonary PCM. This finding is similar to that described for allergic bronchopulmonary aspergillosis where Treg cells contribute to the exacerbated Th2 response and deleterious eosinophilic inflammation^42^.

A recently described suppressor cytokine, IL-35, is produced by nTregs and capable of inducing Tr35 cells that do not express Foxp3. Levels of IL-35 were decreased in the lungs of DT-treated mice. Since nTregs are natural sources of IL-35 and their production increases five- to ten-fold upon contact with T cells^43^, the decrease of this cytokine during Treg cell depletion is aligned with our previous findings demonstrating that *P. brasiliensis* infection induces the migration of nTreg cells to the lungs of mice^22^. Among the cytokines analyzed in this study, an expressive increase in IFN-γ, a characteristic Th1 cytokine, was detected at the early post-infection period analyzed. During *M. tuberculosis* infection, the depletion of the Treg cells has also induced a higher production of IFN-γ and a significant decrease in the bacterial loads when compared to the control mice^44^. In pulmonary PCM, Cano *et al*.^45^ demonstrated that in both susceptible B10.A and resistant A/J mouse strains the neutralization of endogenous IFN-γ resulted in exacerbation of lung infection, early dissemination to the spleen and liver, and deficiency in the protective immune response.

In addition to CD4^+^ IFN-γ^+^ T cells, increased presence of CD8^+^IFN-γ^+^ T lymphocytes were detected in the lungs of DT-treated mice at both post-infection periods. This result agrees with our previous studies demonstrating that aside from CD4^+^ Th1 cells, resistance to *P. brasiliensis* infection is mediated by CD8^+^IFN-γ^+^ T lymphocytes^46^. In this scenario, the actions of IL-12 and IL-23 cytokines are equivalent, since both are able to increase proliferation of memory Th1 cells, as well as the production of IFN-γ^47^. Another important observation was the increased differentiation of CD4^+^ T cells to the Th17 profile mediated by Treg cells depletion. This T cell subpopulation, that is associated with immunoprotection and the less severe forms of PCM^16,21,22^, can be induced by TGF-β signaling which promotes the expression of Foxp3 transcription factor and directs the differentiation of naïve CD4^+^ T cells to the Treg phenotype^48^. IL-23 and IL-6, however, prevent the induction of Foxp3 by TGF-β, and lead to the differentiation of naïve T cells into the Th17 subset via the induction of the Rorc transcription factor^49^.

Over the past years regulatory T-cells with Th17 characteristics have been described^50^. Recently, CD4^+^Foxp3^+^ Treg cells with a pro-inflammatory Th17-like phenotype were also found in cultures of *A. fumigatu*s-stimulated human peripheral blood mononuclear cells^51^. An equivalent finding was also described in pulmonary PCM. In our previous study^22^ using SCID mice adoptively transferred with naive CD4^+^, Treg, or naive CD4^+^ plus Treg cells, we could verify that the transfer of Tregs alone was sufficient to induce a small, but significant, protection. This finding was associated with the expansion of some CD4^+^IL17^+^ T cells and increases expression of RORc, suggesting that some transferred Tregs were also expressing IL-17. The Treg depletion here reported could lead, at least theoretically, to reduced Foxp3^+^IL-17^+^ T-cells, and reduced protection of hosts. However, at both periods assayed, a less severe disease was observed, indicating that this double-positive phenotype cells have a minor contribution to PCM immunoprotection.

It is important to note that the increased Th17 response here described was not associated with an exacerbated inflammation in any of the target organs, as demonstrated by the histopathological analysis. In contrast, the expansion of Th17 cells mediated by decreased presence of Treg cells in disseminated candidiasis caused an exacerbated inflammatory pathogenic response. However, in other forms of the disease, such as gastrointestinal candidiasis, the expansion of Th17 lymphocytes results in decreased colonization by *C. albicans*^52^. Although the effects attributed to the IL-23/Th17 axis may be positive or negative, in systemic fungal infections the absence or the defective differentiation of Th17 cells has usually been associated with negative effects such as recurrent pneumonia due to filamentous fungi and the occurrence of mucocutaneous candidiasis^53,54^. In agreement, our results suggest a protective Th17 response mainly expanded at the late phase of the infection.

Another benefit related to the depletion of Treg cells in ongoing PCM was the decreased mortality observed in DT-treated DEREG mice. This finding was concomitant with the improved cellular immunity observed, which possibly caused a more efficient eradication of the pathogen and decreased tissue pathology. An equivalent response was found in the infections by *P. yoelii, Leishmania spp*. and *M. tuberculosis*^36–38^.

We believe that our findings are important because the reduction of Treg cells could be used as a new immunotherapy for PCM that is typically diagnosed late, when the infection is well established. Indeed, targeting a CTLA-4 blockade with an anti-CTLA-4 monoclonal antibody to reduce Treg activity has previously been used as an immunotherapy of cancer^55^ and infectious diseases^56^. Another possibility would be the use of agonists or antagonists of the indoleamine 2,3 dioxygenase/aryl hydrocarbon receptor (IDO/AhR) axis that is an important inducer of Treg cells in murine PCM^21,57–59^. We believe that new therapeutic approaches are particularly important to PCM patients whose treatment requires intensive and prolonged antifungal chemotherapy that causes deleterious side effects^60^.

In conclusion, using a model that allows for the proper manipulation of Treg cells *in vivo* we demonstrated that the reduction of this T cell subpopulation is highly beneficial to the established PCM because it restores immunity and reverses disease severity. Moreover, this finding introduces the possibility of using Treg cells reduction as a novel immunotherapeutic tool.

## Materials and Methods

### Ethics statement

Animal experiments were performed in strict accordance with the Brazilian Federal Law 11,794 establishing procedures for the scientific use of animals, and the State Law establishing the Animal Protection Code of the State of São Paulo. All efforts were made to minimize suffering, and all animal procedures were approved by the Ethics Committee on Animal Experiments of the Institute of Biomedical Sciences of University of São Paulo (Proc.180/2011-CEEA).

### Mouse strains

C57BL/6^DTR/eGFP^ (DEREG) mice transgenic for the expression of diphtheria toxin receptor (DTR) and GFP (“green fluorescent protein”) under the influence of FoxP3 transcription factor gene, which is characteristic of Treg cells, were here employed. In some experiments, wild type (WT) C57BL/6 mice were used as control. WT and DEREG C57BL/6 mice obtained from Jackson Laboratories were bred and maintained at the University of São Paulo animal facilities under specific-pathogen-free (SPF) conditions in microisolator cages. Male mice were used at 6–8 weeks of age and received sterilized rodent chow and water *ad libitum*.

### Fungus and infection

The highly virulent *P. brasiliensis* 18 isolate (Pb18) was used throughout this study. Yeast cells were maintained by weekly sub-cultivation in semisolid Fava Netto culture medium^61^ at 36 °C and used on days 5–7 of culture. For infection studies, fungal particles were washed in phosphate buffered saline (PBS), counted and adjusted to 20 × 10^6^ cells mL. The viability of fungal suspensions, determined by Janus Green B vital dye (Merck), was always higher than 85%. Mice were anesthetized and submitted to intra-tracheal (i.t.) *P. brasiliensis* infection as previously described^62^. Briefly, after intraperitoneal injection of ketamine and xylazine, animals were infected with 1 × 10^6^ Pb18 yeast cells, contained in 50 µL of PBS, by surgical i.t. inoculation.

### Treg cell depletion

Depletion was performed as previously described^24^. DEREG mice were treated with diphtheria toxin (DT) after 3 weeks of infection with 1 × 10^6^ *P. brasiliensis* yeasts. The dose of 0.5 µg of DT in 200 µL of PBS was injected intraperitoneally (i.p.) into each mouse for two consecutive days, once a week. The procedure was repeated every week until the end of the experiment. Control infected DEREG mice were treated with 200 µL of PBS at the same post-infection periods. DT was purchase from Sigma and reconstituted according to the manufacturer’s protocol.

### Evaluation of Autoimmunity

Uninfected DEREG mice were treated i.p. with 0.5 µg of DT in 200 µL of PBS or just PBS (control) for two consecutive days once a week for 10 weeks. The mice were observed each other day and were euthanized at the tenth week of treatment. After perfusion with 2% paraformaldehyde (PFA) via the aortic route, the target organs (lung, liver, spleen, brain, heart and kidneys) were removed and analyzed by histology. The organs were preserved in Carnoy fixative at room temperature. After the paraffin inclusion step, the organs were cut to a thickness of 5 mm and stained with hematoxylin-eosin (HE), for cellular and tissue identification and analysis.

### Colony forming units (CFU) assays

To assess the viable number of CFU in target organs, lungs, livers and spleens from DT- treated and untreated DEREG mice were aseptically removed, weighted and homogenized in 5 mL PBS using tissue grinders as previously described^63^. Next, 100 µL aliquots of 50- and 100-fold dilutions from organs were plated onto petri dishes containing brain heart infusion agar (Difco) supplemented with 5% *P. brasiliensis* 192 culture filtrate and 4% (v/v) horse serum (Instituto Butantan, São Paulo, Brazil), and incubated at 36 °C. Colonies were counted until no increase in counts was observed and CFU per gram of tissue were determined.

### Mortality rates

Mortality studies were performed with DT or PBS treated DEREG mice inoculated i.t. with 1 × 10^6^ yeast cells. Deaths were registered daily and the mean survival time after infection was calculated.

### Histopathological analysis

Lungs, liver and spleen from DT or PBS treated DEREG mice were collected at weeks 6 and 10 post-infection, fixed in 10% formalin and embedded in paraffin. Sections of 5 µm were stained with hematoxilin-eosin (H&E) for analysis of the lesions and Grocott for fungal evaluation. Pathology was analyzed based on the size, morphology and cell composition of granulomatous lesions, presence of fungi and intensity of the inflammatory infiltrates. Morphometric analysis was performed using a Nikon DXM 1200c digital camera and Nikon NIS Elements AR 2.30 software. Results are expressed as the mean ± standard deviation (SD) for the total area of lesions.

### Flow cytometry analysis

Lung cell suspensions were prepared as previously described^64^. For cell-surface staining, leukocytes were washed and resuspended at 1 × 10^6^ cells/mL in staining buffer (PBS, 2% fetal calf serum and 0.1% NaN_3_). Fc receptors were blocked by the addition of unlabeled anti-CD16/32 (Fc block; BD Biosciences). Leukocytes were then stained in the dark for 20 min at 4 °C with the optimal dilution of each monoclonal antibody: Brilliant Violet 510 (BV)-labeled anti-CD45; Pacific Blue (PB)-labeled anti-CD4; phycoerythrin (PE)-labeled, anti-CTLA-4, anti-IL-17, PE-Cy5-labeled anti-CD69; PECy7-labeled anti-CD25, anti-IL-4; peridinin chlorophyll protein (PerCP)-labeled anti-CD25, anti-ICOS, anti-IFN-γ; allophycocyanin (APC)-labeled anti-CD8, anti-GITR, anti-F4/80; APC-Cy7-labeled anti-CD4, fluorescein isothiocyanate (FITC)-labeled anti-Foxp3 (from BD Biosciences or BioLegend),. Cells were washed twice with staining buffer, fixed with 1% paraformaldehyde (Sigma) and acquired using a FACSCanto II equipment and FACSDiva software (BD Biosciences). For intracellular detection of cytokines, leukocytes obtained from lungs were stimulated for 6 hours in complete RPMI medium containing 50 ng/mL phorbol 12-myristate 13-acetate, 500 ng/mL ionomycin (Sigma-Aldrich), and 3 mM monensin (eBioscience). Next, cells were labeled for surface molecules and then treated according to the manufacturer’s protocol for intracellular staining using the Cytofix/Cytoperm kit (BD Biosciences). Cells were washed twice with staining buffer, resuspended in 100 μL, and an equal volume of 2% formalin was added to fix the cells. A minimum of 50,000 events were acquired on FACScanto II flow cytometer (BD Biosciences) using the FACSDiva software (BD Biosciences). Lymphocytes were gated as judged from forward and side light scatter. For Treg cell characterization, FACS plots or histograms were gated on live CD4^+^FoxP3^GFP+^ cells. Otherwise, gated cells were measured for CD4 or CD8 expression and then for the respective surface or intracellular molecules.

### RNA isolation and cDNA synthesis

Lungs were homogenized in TRIzol reagent using tissue grinders. Phase separation was achieved following addition of 0.2 mL chloroform per mL of TRIzol and centrifugation at 12000 × *g* for 15 min at 4 °C. The upper aqueous RNA phase was transferred to a fresh tube and further purified using Ultraclean Tissue & Cells RNA Isolation Kit (MO BIO Laboratories) according to the manufacturer’s protocol. RNA purity and concentration were assessed on a NanoDrop ND-1000 spectrophotometer. An amount of 1 µg total RNA was reverse transcribed in a 20 µL reaction mixture using the High Capacity RNA-to-cDNA kit (Applied Biosystems) following the manufacturer’s instructions.

### Real-time quantitative polymerase chain reaction (RT–PCR)

The cDNA was amplified using TaqMan Universal PCR Master Mix (Applied Biosystems) and predeveloped TaqMan assay primers and probes (Tbet, Mm00450960_m1; GATA3, Mm00484683_m1; RORγC, Mm01261022_m1; Foxp3, Mm00475162_m1; IL-17, Mm00439618_m1; IL-4, Mm00445260-m1; GAPDH, Mm99999915_g1; all from Applied Biosystems). Probes were labelled with 6-carboxyfluorescein (FAM) at their 5′-terminal end. Data were normalized to GAPDH gene expression. *Taq*Man PCR assays were performed on a MxP3000P QPCR System and data were developed using the MxPro QPCR software (Stratagene).

### Cytokine detection

Lungs from mice infected with *P. brasiliensis* were aseptically removed and individually disrupted in 5 mL of PBS. Supernatants were separated from cell debris by centrifugation at 3000 × *g* for 20 min and stored at −80 °C. The levels of IL-2, IL-4, IL-6, IL-10, IL-12, IL-17, IL-23, IL-1β, IFN-γ TNF-α, IL-35 and TGF-β were measured by capture enzyme-linked immunosorbent assay (ELISA) with antibodies pairs purchased from eBioscience. The ELISA procedure was carried out according to the manufacturer’s protocol. Plates were read using a spectrophotometric plate reader (VersaMax, Molecular Devices).

### Statistics

Data are expressed as the Mean ± SD. The omnibus K2 D’Agostino-Pearson normality test was used to test the Gaussian distribution of samples. Differences between groups were analyzed by non-paired Student’s *t* test or analysis of variance (ANOVA) followed by the Tukey test. Differences between survival times were determined with the LogRank test. Data were analyzed using GraphPad Prism 5.01 software (GraphPad Prism Software, Inc.). *p* values ≤ 0.05 were considered significant.

## Electronic supplementary material

Supplementary Information

## Data Availability

The datasets generated during and/or analyzed during the current study are available from the corresponding author on reasonable request.
