# Supplementary material for: Depletion of regulatory T cells in ongoing paracoccidioidomycosis rescues protective Th1/Th17 immunity and prevents fatal disease outcome
Source: Sci Rep. 2018 Nov 8;8:16544. doi: 10.1038/s41598-018-35037-8 (PMC6224548; doi:10.1038/s41598-018-35037-8)
Supplement: Supplementary file 1 — Supplementary Information [file 41598_2018_35037_MOESM1_ESM.pdf]

SUPPLEMENTARY INFORMATION

Depletion of regulatory T cells in ongoing paracoccidioidomycosis rescues protective Th1/Th17 immunity and prevents fatal disease outcome

Nayane A L Galdino, Flávio V Loures, Eliseu F de Araújo, Tania A da Costa, Nycolas W Preite and Vera Lúcia G Calich*

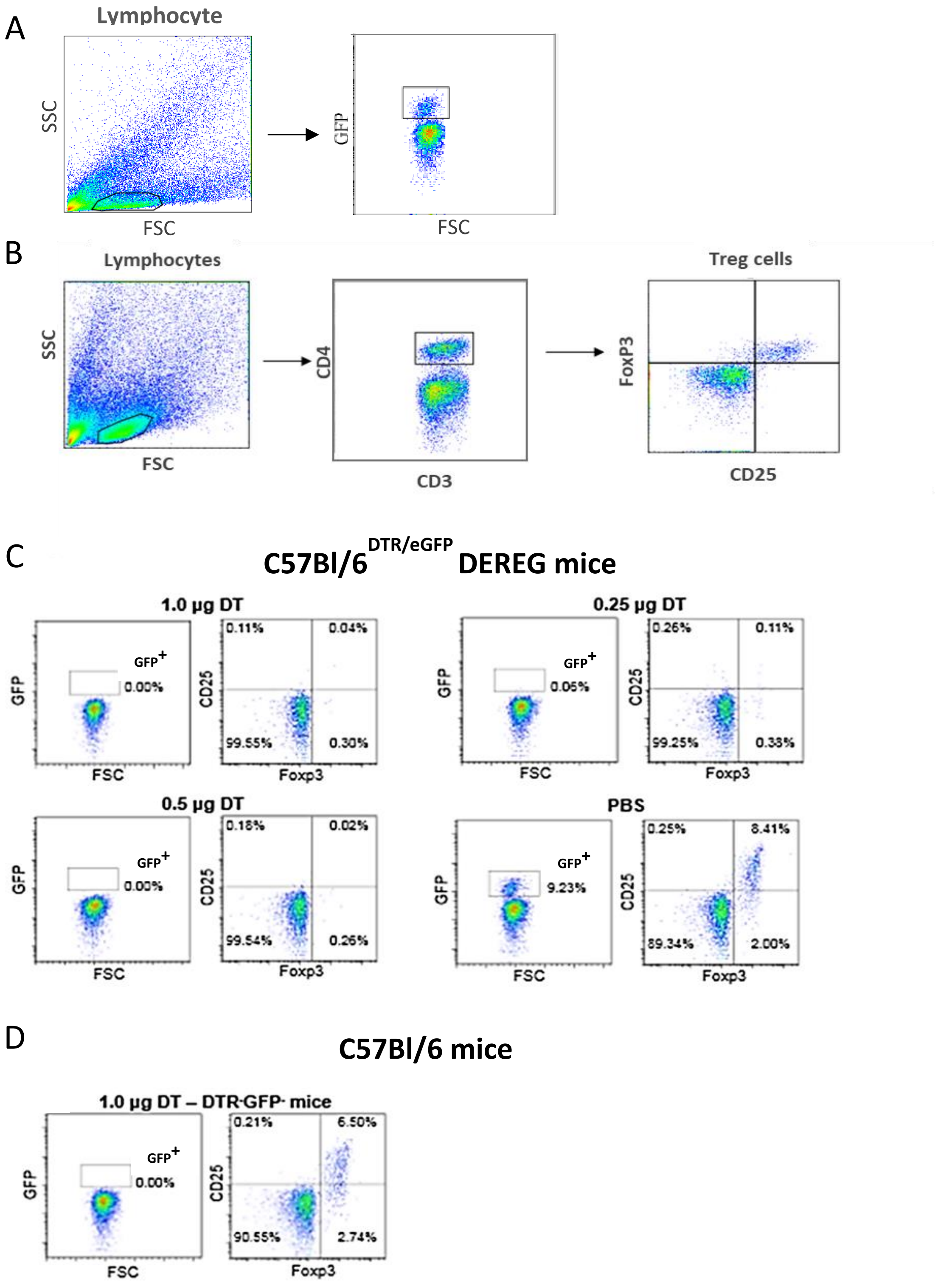

S1 Fig DT titration for Treg cells depletion in DEREG mice. Doses of 1.0, 0.5 or 0.25 µg of diphtheria toxin (DT) or PBS were injected i.p. for two consecutive days in C57BL/6$^{DTR/eGFP}$ (DEREG) mice that were sacrificed 12 h later. Lung leukocytes were obtained and labelled with anti-CD25 and anti-Foxp3 antibodies and the frequency of GFP^{+} cells and CD25^{+}Foxp3^{+} were determined by flow cytometry using two different gate strategies. **A**- Gate strategy used to characterize the frequency of GFP^{+} Treg cells. Lung lymphocytes were first identified by FSC X SSC analysis and Treg cells by GFP expression. **B**-Gate strategy to identify CD25^{+}Foxp3^{+} Treg cells. Lung lymphocytes were first identified by FSC X SSC analysis, CD4^{+} lymphocytes trough CD4 and CD3 expression. Within the CD4^{+} population, the number of Treg cells was quantified as the percentage of CD25^{+}Foxp3^{+} cells. **C**-The doses of 1.0 and 0.5 µg of DT induced a robust depletion of Tregs. **D**- As control, doses of 1.0 µg of DT were injected in C57BL/6 WT mice and no Treg cells depletion was seen.

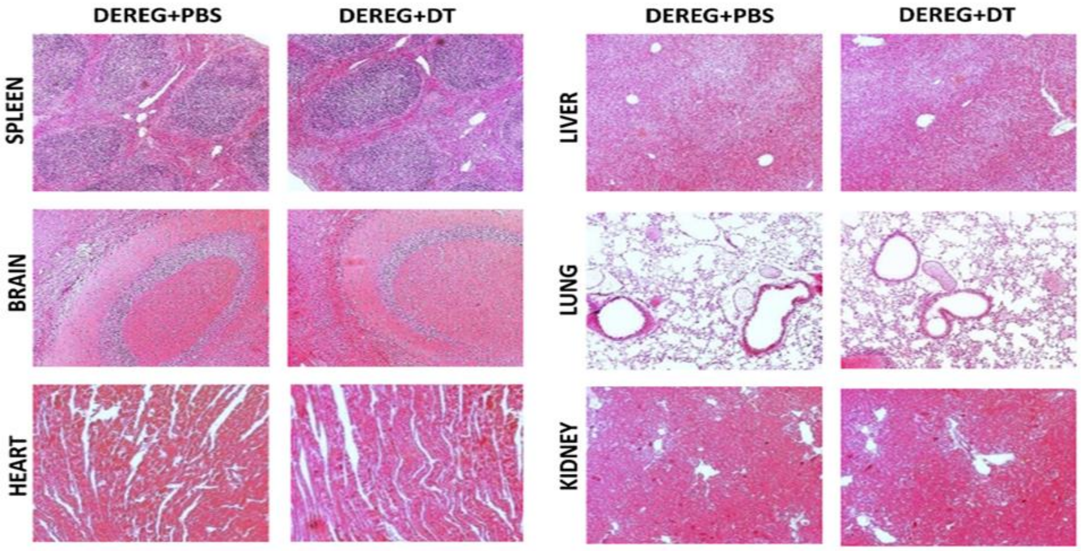

S2 Fig Treg cells depletion does not cause autoimmunity in DEREG mice. DEREG mice were treated during 10 weeks with two consecutive daily doses/week of 0.5 µg of DT by the i.p. route. At week 10, mice were euthanized their organs (lung, liver, spleen, brain, heart and kidneys) removed, fixed, cut (5 µm) and stained with hematoxylin-eosin (HE). The organs were analyzed using a Nikon microscope and no inflammatory infiltrates were observed in any analyzed organs indicating absence of auto-immunity in Treg depleted mice (n=6/group).

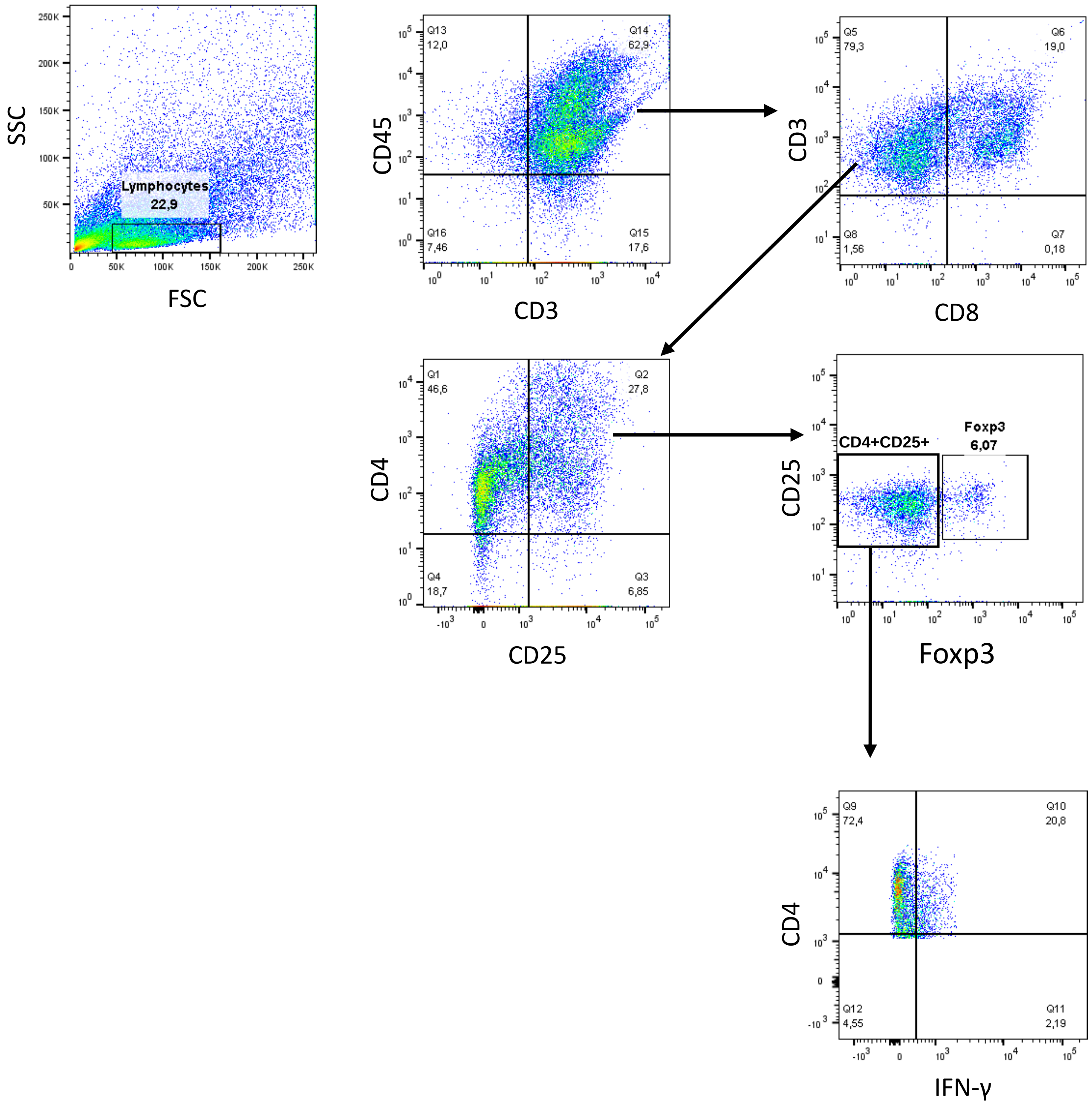

S3 Fig Gate strategy used to phenotype CD4$^+$CD25$^+$Foxp3$^+$ Treg and cytokine expressing CD4+ T cells. Lung leukocytes were obtained, washed and resuspended at 1×10^6 cells/mL in staining buffer. Fc receptors were blocked by the addition of unlabeled anti-CD16/32. Leukocytes were then stained in the dark for 20 min at 4°C with the optimal dilution of each monoclonal antibody. Cells were washed twice with staining buffer, fixed with 1% paraformaldehyde (Sigma) and acquired using a FACSCanto II equipment and FACSDiva software (BD Biosciences).

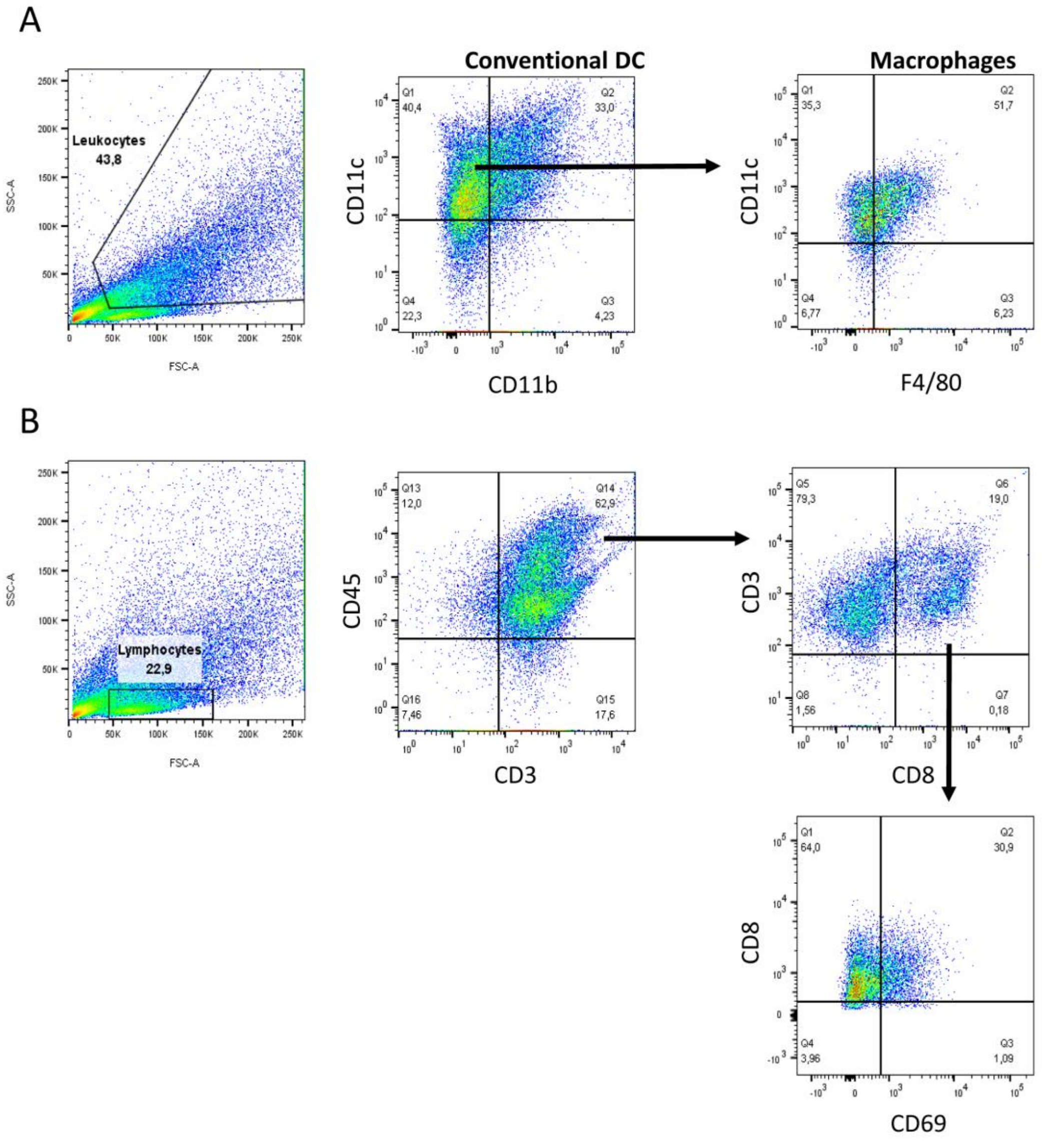

S4 Fig Gate strategy used to phenotype macrophages ($CD11c^+F4/80^+$) and activated $CD8^+$ cells ($CD8^+CD69^+$). Lung leukocytes were obtained, washed and resuspended at 1×10^6 cells/mL in staining buffer. Fc receptors were blocked by the addition of unlabeled anti-CD16/32. Leukocytes were then stained in the dark for 20 min at 4°C with the optimal dilution of each monoclonal antibody. Cells were washed twice with staining buffer, fixed with 1% paraformaldehyde (Sigma) and acquired using a FACSCanto II equipment and FACSDiva software (BD Biosciences). (A) Gate strategy for $CD11c^+F4/80^+$ macrophages, and (B) for $CD8^+CD69^+$ lymphocytes.

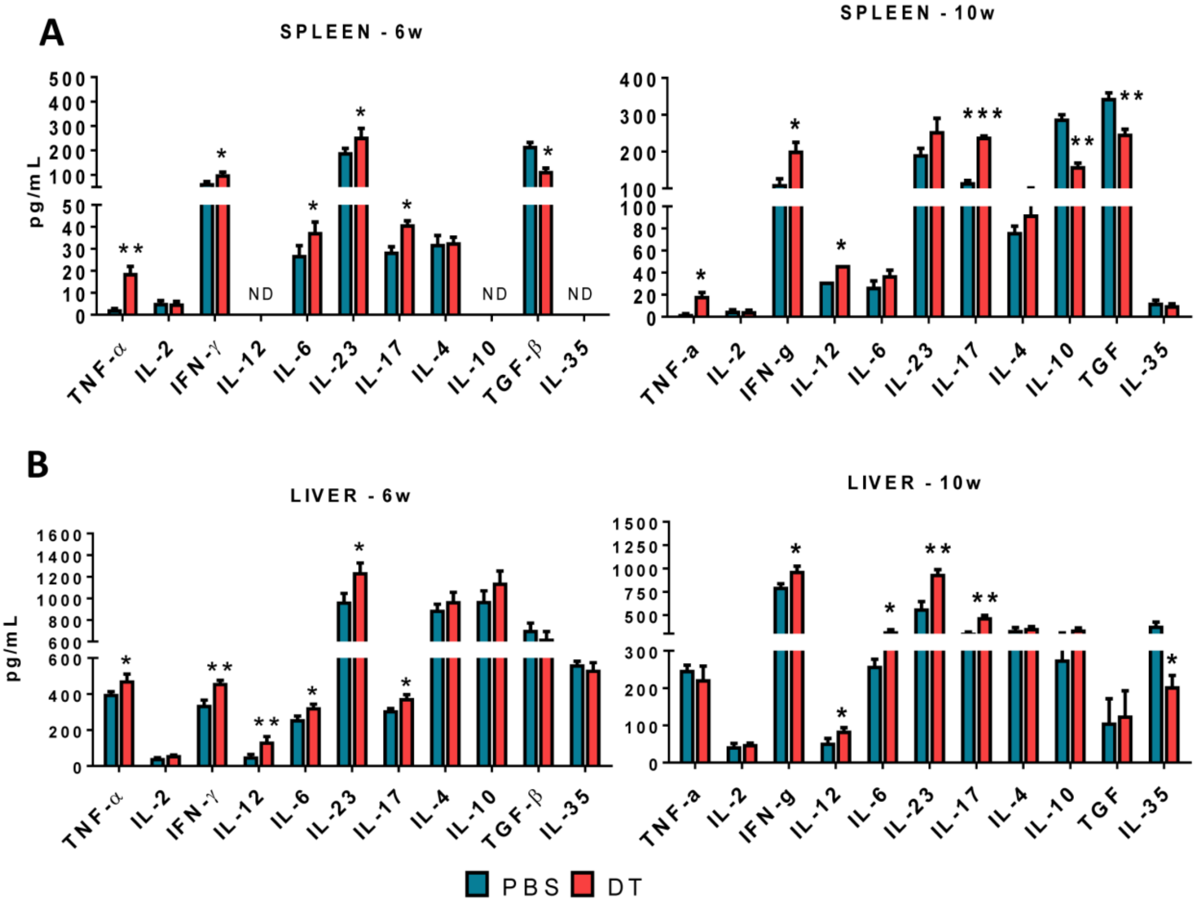

S5 Fig The reduction of Treg cells in ongoing PCM of DEREG mice controls the levels of hepatic and splenic cytokines. Cytokines were measured by ELISA in the spleen (A) and liver (B) homogenates of DT and PBS (control) treated DEREG mice at weeks 6 and 10 following *P.brasiliensis* infection. Bars show mean ± SEM of two independent experiments using 5 mice per group (* $p<0.05$, ** $p<0.01$).
